# Differential Analysis of Fecal SCFAs and Their Contribution to Adipogenesis in UCP1 Knock-In Pigs

**DOI:** 10.3390/vetsci12020102

**Published:** 2025-02-01

**Authors:** Chengyu Zhao, Jianfei Pan, Yanfang Wang, Jianguo Zhao, Jiaojiao Huang

**Affiliations:** 1 College of Animal Science and Technology, Qingdao Agricultural University, Qingdao 266109, China; zcy@stu.qau.edu.cn; 2 College of Life Science, Qingdao Agricultural University, Qingdao 266109, China; 3 State Key Laboratory of Animal Biotech Breeding, Institute of Animal Science, Chinese Academy of Agricultural Sciences, Beijing 100193, China; pjf124124@gmail.com (J.P.); wangyanfang@caas.cn (Y.W.); 4 State Key Laboratory of Stem Cell and Reproductive Biology, Institute of Zoology, Chinese Academy of Sciences, Beijing 100101, China; zhaojg@ioz.ac.cn

**Keywords:** pigs, *UCP1*, SCFAs, caproic acid, fat deposition, gut microbiota

## Abstract

This study aimed to investigate the changes in fecal short-chain fatty acids (SCFAs) content in UCP1 knock-in pigs (KI pigs) and their effect on adipogenesis. Fecal samples from five 6-month-old wild-type (WT) and KI pigs were collected for targeted metabolomics and 16s rRNA sequencing analyses to identify differences in SCFAs and gut microbiota that may contribute to regulating fat deposition in pigs. The metabolome of pig fecal samples targeted for an analysis of SCFAs identified seven SCFAs, with caproic acid (except isovaleric acid) being the significantly different one. The results of the fecal 16s rRNA analysis demonstrated a notable reduction in the abundance of *Streptococcus* spp. in the KI pigs in comparison to the WT pigs, with a statistically significant difference. Correlation analyses demonstrated a statistically significant positive correlation between the abundance of *Streptococcus* spp. and SCFAs, as well as pig body weight and fatness. It was postulated that the reduction in SCFAs in the intestinal tracts of KI pigs may be associated with a reduction in *Streptococcus* spp. abundance. Compared to WT pigs, the concentration of fecal SCFAs in KI pigs was significantly reduced, which may be related to the decreased abundance of *Streptococcus*. The in vitro experiments showed that caproic acid could significantly enhance the differentiation efficiency of porcine SVF cells into mature adipocytes by activating the *FFAR4* gene.

## Simple Summary

Short-chain fatty acids (SCFAs) are the primary metabolites produced by the gut microbiota from the fermentation of dietary fibre. They frequently function as a kind of signal molecule, regulating the expression of genes within the host cell. The results of animal studies indicate that SCFAs are involved in the acquisition of energy and may contribute to the development of obesity. In this study, we utilized targeted metabolomics and 16s rRNA sequencing techniques to pinpoint gut microbial communities potentially implicated in short-chain fatty acid metabolism. The identified short-chain fatty acids were then applied in in vitro adipocyte validation and lipogenic function studies. These efforts aim to offer a scientific foundation for the commercialization of *UCP1* gene-edited pigs.

## 1. Introduction

China is a populous country with a high level of meat consumption. Pork is the primary meat food for both urban and rural residents, accounting for approximately 67% of all meat consumption. Consequently, the cultivation of pig breeds with a high growth rate and high leanness has consistently been a key objective in pig breeding. The identification of candidate genes, gut microbiota, and metabolites associated with fat deposition has emerged as a pivotal research area. In a previous study, we generated genetically modified pigs (hereinafter referred to as KI pigs) with adipose tissue-specific expressions of the *UCP1* gene using CRISPR/Cas9 technology. The results demonstrated that the KI pigs exhibited a markedly enhanced thermoregulatory capacity in comparison to the wild-type (WT) pigs (*p* < 0.01). Additionally, they displayed a notable reduction in adiposity (4.89%), a considerable decrease in backfat thickness (2.4 mm), and a substantial increase in lean body mass (3.38%). Consequently, the KI pigs exhibited a low adiposity and a high lean body mass, a trait of significant commercial value [1]. While previous studies have elucidated that the expression of the *UCP1* gene in adipose tissue promotes lipolysis, further clarification is required regarding the specific molecular mechanisms underlying the low fat rate observed in KI pigs.

The intestinal flora represents a significant component of the normal ecosystem of the host, comprising thousands of distinct microorganisms within the gut, collectively referred to as the ‘second genome’ [2]. Over an extended period of evolutionary history, the host organism and its associated microbiota have co-evolved to form a mutually beneficial symbiotic relationship. This relationship is involved in a range of physiological and pathological processes, including digestion, absorption, immunity, and the development of disease in the host [3]. The composition of microorganisms is subject to a number of factors, including dietary intake, environmental conditions, and genetics [4]. Wu et al. investigated the effects of HIF-2α deficiency on the intestinal microbiota using a mouse model of intestinal epithelial cells. They observed that *HIF-2α* deficiency altered the intestinal microbiota, reduced lactate synthesis, increased the abundance of *Ruminococcus* torques in the intestine, and upregulated intestinal and plasma deoxycholic acid levels. This resulted in the activation of the white adipose tissue expression of *TGR5* to promote thermogenesis and the amelioration of high-fat diet-induced obesity [5]. In our preceding study, we discovered that *UCP1* KI pigs exhibited markedly enhanced thermoregulatory capabilities compared to wild-type (WT) pigs. Additionally, we observed a notable increase in lean body mass. Subsequent analysis revealed that plasma free fatty acid (FFA) levels were significantly elevated, while triglyceride content was significantly reduced in KI pigs. Subsequent lipidomic investigations of adipose tissue from KI pigs revealed that the adipose-specific expression of *UCP1* markedly altered the lipid composition of inguinal subcutaneous adipose tissue from Parma pigs, with a notable reduction in triglycerides and an increase in phospholipids and sphingolipids [6]. Consequently, the modified lipid constitution of adipose tissue can result in alterations in lipids within the organismal system, thereby regulating host gut microbiota homeostasis. Subsequent findings demonstrated that *UCP1* expression not only reduced the expression of genes closely related to bile acid metabolism but also resulted in alterations to the composition of the intestinal flora. This was accompanied by increased levels of porcine deoxycholic acid in vivo and collectively facilitated the hydrolysis of adipose tissue [7].

The colon represents a significant site of microbial fermentation. Nutrients in foods that are not fully digested are transported to the colon, where they are broken down by the gut microbiota. It has been demonstrated that in excess of one-third of the small molecules present in the blood of humans originate from the microbial flora of the gut [8,9], The metabolites of gut microorganisms can be absorbed into the bloodstream via the hepatic–intestinal circulation, thereby participating in the regulation of host physiological processes [10]. Short-chain fatty acids (SCFAs), including acetic, propionic, butyric, and valeric acids, are the primary metabolites produced by intestinal bacteria from the breakdown of dietary fibre. As microbial derivatives that are highly abundant in the intestinal lumen, SCFAs are not only metabolic by-products, but they also act as signal molecules that can activate gene expression in host cells. This action helps to alleviate and regulate key physiological processes, including intestinal inflammation, the nervous system, and the endocrine system. For example, butyrate and other SCFAs have been shown to promote B-cell differentiation and increase the number of B-cells. Additionally, butyrate has been demonstrated to inhibit the secretion of pro-inflammatory factors and chemokines by neutrophils in patients with enteritis, thereby attenuating the intestinal inflammatory response [11,12]. The compound activates the intestinal AMPK signal pathway, which in turn increases the expression of genes related to Occludin, Claudins, and Cadherin in the intestinal tight junction (TJ) proteins. This has the effect of regulating intestinal permeability and the integrity of the intestinal barrier, thereby promoting intestinal functions [13,14]. Furthermore, SCFAs have been demonstrated to stimulate the release of cholecystokinin, thereby activating the vagus nerve and increasing feelings of satiety [15]. Short-chain fatty acids (SCFAs) within the colon bind to G protein-coupled receptors (GPCRs), thereby stimulating the production of gut hormones, including glucagon-like peptide-1 (GLP-1) [16]. The results of animal studies indicate that SCFAs may play a role in energy acquisition, which in turn contributes to the development of obesity [17]. Nevertheless, it remains unclear whether there are alterations in the SCFAs present in the intestines of UCP1 pigs, and whether these altered SCFAs are involved in the deposition of fat in pigs.

In this study, targeted metabolomic techniques were employed to examine the abundance and fractions of short-chain fatty acids (SCFAs) in fecal samples from adult wild-type (WT) and UCP1 knock-in pigs. Furthermore, the gut microbial communities potentially involved in SCFA metabolism were analyzed in depth by 16s rRNA sequencing. Furthermore, in vitro adipocyte differentiation and lipogenic function studies were conducted on the screened differential SCFAs, which contributed to our in-depth understanding of the molecular mechanisms underlying the low adiposity observed in UCP1 pigs. This study not only enhances our comprehension of the regulatory mechanisms of porcine fat metabolism but also furnishes a scientific foundation for the commercialization of *UCP1* gene-edited pigs.

## 2. Materials and Methods

### 2.1. Animal Material and Sample Collection

In this experiment, fecal samples were collected from five Bama pigs of each of the following two genotypes: wild-type (WT) and *UCP1* gene-edited (KI) [1]. The pigs were at peak growth (6 months of age) and were maintained under uniform feeding conditions (on standard diets and free water). Upon the collection of the fecal samples, the fresh feces were immediately placed in liquid nitrogen and subsequently transported back to the laboratory, where they were stored at −80 °C. All experimental animals were housed at the Northern Large Animal Research Base of the Institute of Zoology, Chinese Academy of Sciences, and the animal experimental operations were approved by the Animal Ethics Committee of the Institute of Zoology, Chinese Academy of Sciences.

### 2.2. The 16s rRNA Assay and Data Analysis

The extraction of DNA from fecal samples, PCR amplification, product recovery, library construction, sequencing, data uptake, and analysis were all conducted by Shanghai Paiseno Bio-technology Co., Ltd. (Shanghai, China). The genomic DNA of the fecal samples was extracted using the Tengen Fecal Extraction Kit [7]. Two-by-two comparisons of the relative abundance at the phylum and genus levels of WT and KI pigs at different time points were tested for the significance of differences using the Kruskal–Wallis function in R. The resulting *p*-values were adjusted for multiple comparisons using the Holm method, and *p*-values less than 0.05, 0.01, and 0.001 were considered statistically significant. A principal component analysis (PCA) was conducted using the R software, employing the ‘gmodels’ function. A heatmap was generated with the ‘pheatmap’ function in R. Prior to conducting the PCA and heatmap, the data underwent a z-score calculation between groups, which removed the potential for interference with the display of results due to the considerable differences in lipid content or gene abundance.

### 2.3. Determination of Short-Chain Fatty Acids

The determination of fecal short-chain fatty acids was conducted in accordance with the following procedure: the sample was thawed at a slow rate at 4 °C; 1 mL of a pre-cooled methanol/acetonitrile/water solution with a volume ratio of 2:2:1 was added, vortexed, and mixed, and then subjected to ultrasound treatment. The sample was incubated at 4 °C for 30 min, placed at −20 °C for 10 min, and subsequently centrifuged at 14,000 rpm at 4 °C for 20 min. The supernatant was transferred to a new tube and subsequently dried under vacuum. Another 100 μL of the acetonitrile aqueous solution (volume ratio 1:1) was added to dissolve the sample again. Then the mixture was vortex-mixed and centrifuged at 14,000× *g* for 15 min at 4 °C. Finally, the resulting supernatant was taken for online mass spectrometry analysis. The samples were subjected to ultra-performance liquid chromatography (UPLC) and subsequently analyzed by mass spectrometry (MS) on a Triple TOF 6600 mass spectrometer (ABSCIEX).

### 2.4. Isolation, Culture, and In Vitro Differentiation of Preadipocytes

Porcine precursor adipocytes were isolated from subcutaneous adipose tissue of seven-day-old Parma pigs, minced, and digested with 2 mg/mL type I collagenase (West Sigma, USA) in D-Hanks (Solebo, Beijing, China) balanced salt solution. The cell cultures were incubated in DMEM/F12 (HyClone, Logan, UT, USA) and supplemented with 10% fetal bovine serum (FBS, HyClone) and 1% penicillin–streptomycin (PS, Sigma, Wisconsin, WI, USA). The cultures were maintained at 38.5 °C for 24 h. The complete medium (DMEM/F12 + 10% FBS + 1% PS) was replaced every two days. The cells were allowed to undergo 90% fusion. The cells were allowed to reach 90% confluence, after which they were inoculated in 24-well plates at a density of 0.5 mL and 6 × 10^5^ cells per well. The cells were allowed to reach confluence, after which the standard medium was removed and replaced with the induction medium for a period of five days. On day five, the medium was replaced with half of the maturation medium, and on day six, it was replaced with the mature medium, which was then cultured for a further two days. On day eight, the fully differentiated adipocytes were prepared for the subsequent experiments [18].

### 2.5. Oil Red O Staining

The differentiation and lipogenic efficiency of mature adipocytes were examined through staining with Oil Red O (Solarbio, Beijing, China). Following differentiation for eight days, mature adipocytes were fixed with 4% paraformaldehyde (PFA, Solarbio) for 30 min at room temperature. The cells were then washed with 60% isopropanol and stained with the working solution for 15–20 min at room temperature. After staining, the cells were washed twice with distilled water. Subsequently, the cells were examined microscopically and photographed. The working solution of Oil Red O was prepared according to the following ratio: Oil Red O/deionized water = 4:6, and then filtered at room temperature and stored in a dark location [19].

### 2.6. Extraction and Reverse Transcription of RNA

Total RNA was extracted from cells using the RNAiso reagent (Takara, Osaka, Japan). The purity and concentration of total RNA were determined using a Nanodrop 2000 spectrophotometer (Thermo Fisher Scientific, Waltham, MA, USA). The absorbance ratios (260/280 nm) of all samples were between 1.80 and 2.00, indicating the presence of pure RNA. cDNA was synthesized using the Primer Script RT Reagent Kit with gDNA Eraser (Takara).

### 2.7. Real-Time Fluorescence Quantification (qPCR)

Quantitative polymerase chain reaction (qPCR) was conducted using the Quant Studio 3 system (Thermo Fisher Scientific). The specific primers were designed using the online Primer3 software (version 4.1.0). The qPCR reaction system (20 μL) was prepared as follows: The SYBR-MIX comprised 10 μL, while the Primer-F and Primer-F&R each comprised 0.4 μL. Additionally, 0.4 μL of ROX and 2 μL of cDNA were included, along with 6.8 μL of RNase-free water, resulting in a total volume of 20 μL. Once the aforementioned solutions had been combined, the samples were transferred to 96-well plates, allocated to the appropriate primer and control groups, and then subjected to centrifugation to remove any air bubbles. Following this, the samples were quantified using the appropriate equipment. The PCR amplification procedure was as follows: pre-denaturation, the reaction was initiated at 95 °C for 5 min, followed by denaturation at 95 °C for 5 s, annealing at 60 °C for 34 s, and 40 cycles. The relative expression of genes was calculated using the 2-ΔΔCt method, with 18S serving as the housekeeping gene. The primer information can be found in the attachment “Table S1” [20].

### 2.8. Statistical Analysis

All experiments included a minimum of three biological replicates. All data are presented as means ± standard error of measurement (mean ± SEM). The statistical significance of the differences between the control and experimental groups was evaluated using paired specimen *t*-tests with GraphPad Prism version 8.0.1 software. Pearson Correlation Coefficient was calculated with the “cor” function in the R software. The heatmap was generated by the “pheatmap” function in the R software (R 4.4.1). The data for the heatmap were obtained after being corrected by calculating the z-score between groups, thus eliminating the interference caused by excessive differences in abundances on the result display. The level of significance was defined as follows: *p* > 0.05, no significant difference; * *p* < 0.05, significant difference; ** *p* < 0.01, highly significant difference; and *** *p* < 0.001, highly significant difference [7].

### 2.9. In Vitro Cell Experiment

Inoculate 10^6^ SVF cells derived from Bama pigs into a 12-well plate. Once the cells reach full contact (100% confluence), mark this time point as differentiation—2 days. On day 0, substitute the medium with the induction differentiation medium supplemented with sodium caproate. After 2–3 days, switch to the maintenance medium also containing sodium caproate. Mature adipocytes develop after 8 days of differentiation. The concentrations of sodium caproate in the medium are set at Con, 0.1 mM, 1 mM, and 3 mM.

The induction differentiation medium consists of DMEM medium supplemented with 10% fetal bovine serum (FBS), 1% penicillin–streptomycin (PS), 5 μg/mL Insulin, 0.5 mM 3-isobutyl-1-methylxanthine (IBMX), 0.1 μM Dexamethasone (Dex), and 1 μM Rosiglitazone (Rosi). The maintenance differentiation medium contains DMEM medium with 10% FBS, 1% PS, and 5 μg/mL Insulin.

## 3. Results

### 3.1. The Concentration of Short-Chain Fatty Acids (SCFAs) Is Markedly Diminished in the Feces of KI Pigs

**Figure 1:**
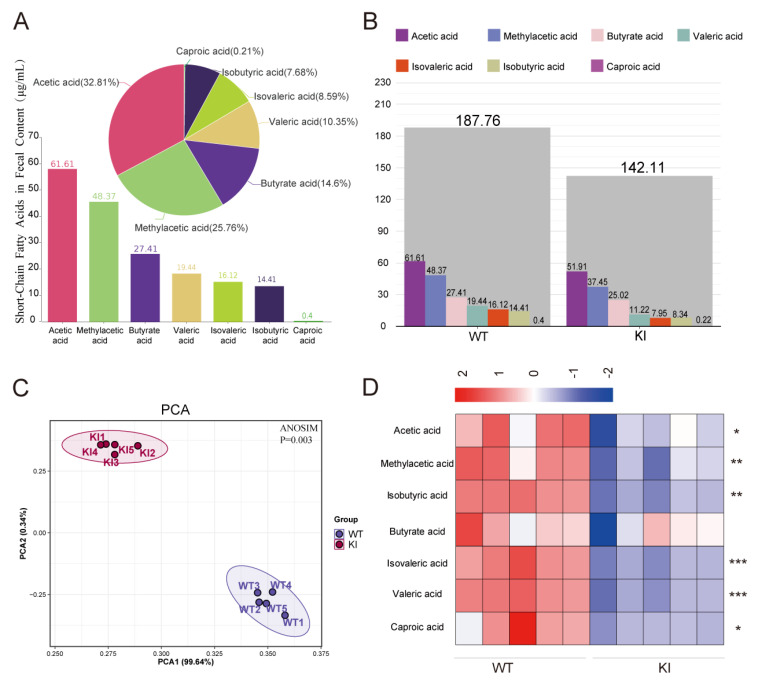
Fecal SCFA contents of KI pigs were significantly lower than those in WT pigs. (**A**) The percentage of fecal SCFAs in WT and KI pigs. (**B**) PCA plot of fecal SCFAs in WT and KI pigs. (**C**) The proportion of fecal SCFAs in WT and KI pigs. (**D**) Heatmap showing differential fecal SCFA levels between WT and KI pigs. * *p* < 0.05, ** *p* < 0.01, *** *p* < 0.001.

In this study, the concentrations of short-chain fatty acids (SCFAs) were quantified in the feces of 6-month-old wild-type (WT) and knock-in (KI) pigs under identical dietary conditions using a targeted metabolomic assay. The results demonstrated the presence of seven SCFAs, including acetic acid, propionic acid, isobutyric acid, butyric acid, isovaleric acid, valeric acid, and caproic acid, in the feces of WT pigs. Among these, acetic acid and propionic acid were the most abundant, representing 32.81% and 25.76%, respectively, while caproic acid was the least prevalent, accounting for only 0.21% (Figure 1A). Subsequently, the SCFAs present in the feces of WT and KI pigs were analyzed to identify any differences. The results demonstrated a notable distinction in the composition of individual SCFAs between the KI and WT pigs, indicating a significant divergence in the SCFA profile between the two groups (*p* < 0.05) (Figure 1B). Additionally, the total SCFA content was found to be significantly lower in the KI pigs compared to the WT pigs (*p* < 0.5) (Figure 1C), and the content of six out of the seven SCFAs in the feces of the two groups was significantly different (Figure 1D), of which caproic acid was also significantly lower in KI pigs than in WT pigs (*p* < 0.05). The content in WT pigs was 1.84 times higher than that in KI pigs (Table 1 and Figure 1B,D).

### 3.2. Distinctive Variations in the Fecal Microbiology of KI and WT Pigs

**Figure 2:**
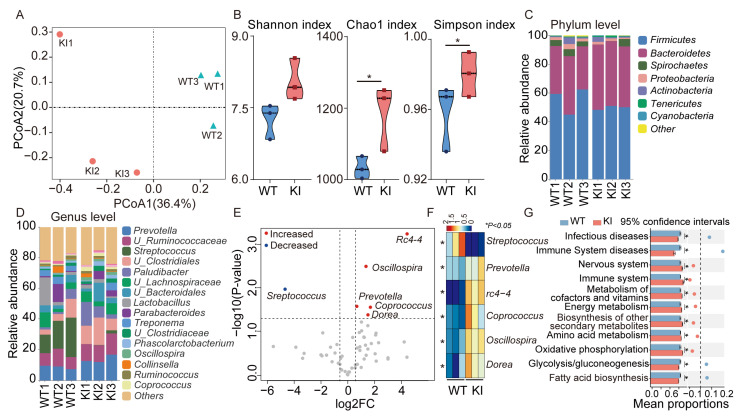
Significant differences in the fecal microbiome between WT and KI pigs. (**A**) PCoA analysis of fecal microbiota from WT and KI pigs. (**B**) Alpha diversity analysis of fecal microbiota from WT and KI pigs. (**C**) Relative abundance of fecal microbiota at the phylum level of WT and KI pigs. (**D**) Relative abundance of fecal microbiota at the genus level of WT and KI pigs. (**E**) The volcano plot shows the differentially abundant genera between WT and KI pigs. (**F**) The heatmap shows the differences in genus abundance between WT and KI pigs, with differentially abundant genera selected from Figure E. (**G**) Metabolic functional profile of the microbial community predicted by PICRUSt. * *p* < 0.05.

Short-chain fatty acids (SCFAs) represent the primary products of dietary fibre fermentation within the intestine. To gain further insight into the molecular mechanisms underlying the observed decrease in SCFA content and alteration in fecal fraction proportions in KI pigs, we employed the 16s rRNA technique to characterize the microbial composition in the feces of WT and KI pigs. The results demonstrated significant discrepancies in the microbial composition of the feces of KI and WT pigs (Figure 2A). Additionally, α-diversity indices, including the Chao1 index and Simpson’s index, exhibited a notable elevation in KI pigs compared to WT pigs (*p* < 0.05), indicating a more diverse bacterial flora in the former (Figure 2B). Furthermore, a comparison of the flora composition of WT and UCP1 pigs at the phylum level (Figure 2C) and at the genus level (Figure 2D) revealed significant discrepancies between the two. For instance, at the phylum level, the abundance of Bacteroidetes was higher in KI pigs than in WT pigs, whereas the abundance of Spirochaetes was lower than in WT pigs (Figure 2C). At the genus level, the abundance of Prevotella was higher than in WT pigs, whereas the abundance of *Streptococcus* was lower than in WT pigs (Figure 2D). Furthermore, six genera were identified as significantly different between WT and KI pigs (Fold Change >2, *p* < 0.05), with *Prevotella*, *Coprococcus*, *Dorea*, *Oscillospira,* and *rc4-4* being significantly more abundant in KI pigs than in WT pigs (Figure 2D). Conversely, *Streptococcus* spp. were significantly less abundant than in WT pigs (Figure 2E,F). Furthermore, functional enrichment revealed that the abundance of metabolism-related pathways, including amino acid metabolism, energy metabolism, and the biosynthesis of secondary metabolites, was markedly elevated in KI pigs relative to WT pigs. Conversely, the abundance of disease-related pathways was significantly diminished in KI pigs compared to WT pigs, indicating that the KI pigs may possess a more robust gut flora structure (Figure 2G). Taken together, the results demonstrated that the gut microbiota of KI pigs exhibited notable differences from that of WT pigs in terms of diversity, composition, and functionality.

### 3.3. SCFA Content Is Significantly and Positively Correlated with Porcine Adiposity and Streptococcus spp. Abundance

**Figure 3:**
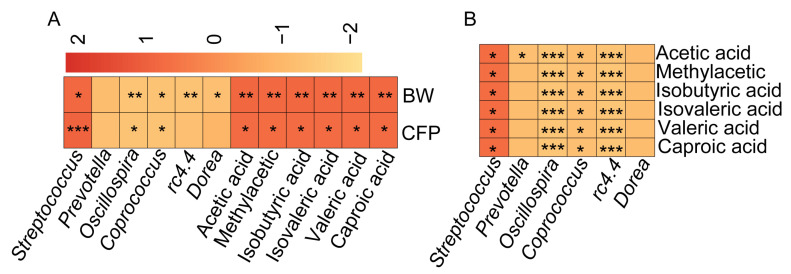
The SCFA contents are significantly positively correlated with fat percentage and the abundance of the *Streptococcus* in pigs. (**A**) Heatmap of the correlation analysis between fecal SCFA contents, the abundance of differential microbiota, and body weight and fat percentage in WT pigs and KI pigs. (**B**) Heatmap of the correlation analysis between fecal SCFAs content and the abundance of fecal differential microbiota in WT pigs and KI pigs; * *p* < 0.05, ** *p* < 0.01, *** *p* < 0.001.

Despite the identification of significant differences in SCFAs (Figure 1) and microbial composition (Figure 2) within the feces of KI pigs compared with those of WT pigs, the inter-relationship between SCFAs and the differential microorganisms, as well as their connection to porcine lipogenesis, remains ambiguous. Consequently, we conducted Pearson correlation analyses of body weight and adiposity in 6-month-old WT and KI pigs with the SCFA content in the feces and differential bacteria. The results demonstrated a significant positive correlation between six differential SCFAs (acetic acid, methylacetic acid, isobutyric acid, isovaleric acid, valeric acid, and caproic acid) and body weight and fat percentage in pigs (Figure 3A). Conversely, the abundance of the differential microorganisms *Prevotella*, *Coprococcus*, and *Oscillospira* exhibited a significant negative correlation with body weight and fat percentage (Figure 3A), while the abundance of *Streptococcus* spp. demonstrated a significant positive correlation with body weight and adiposity, and only *Streptococcus* spp. exhibited a significant positive correlation with the content of SCFAs. In contrast, the abundance of the differentiated bacteria showed a significant negative correlation with the content of SCFAs (Figure 3B). The fat rate of KI pigs was found to be significantly lower than that of WT pigs, and the content of SCFAs in the feces of KI pigs was also significantly lower than that of WT pigs. Correlation analysis demonstrated a significant positive correlation between the two, suggesting that SCFAs may play a role in promoting adipogenesis. Conversely, the abundance of *Streptococcus* spp. was significantly lower in KI pigs than in WT pigs, and it had a significant positive correlation with the fat rate and body weight. It was therefore inferred that the reduction in SCFAs in the intestinal tract of KI pigs might be related to the decrease in the abundance of *Streptococcus*. However, the specific molecular mechanism requires further verification through additional experiments.

### 3.4. Caproic Acid Enhanced the Efficiency of SVF Cell Differentiation to Mature Adipocytes

**Figure 4:**
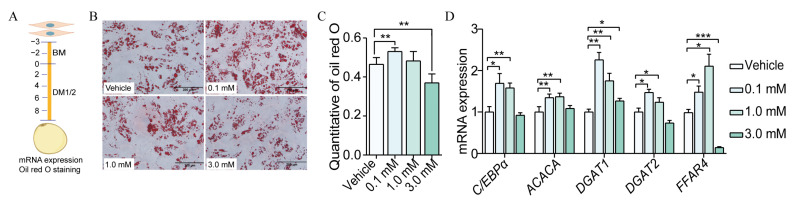
Sodium caprate promotes the efficiency of SVF differentiation into white adipocytes. (**A**) Experimental design diagram of SVF cell induced to differentiate into white adipocytes. (**B**) Perform Oil Red O staining on stromal vascular fraction (SVF) cells that have been induced to differentiate into white adipocytes for 8 days (200 μm). (**C**) Quantification of Oil Red O from 4B; (**D**) the mRNA expression of key adipogenic genes in the control group and sodium caprate treatment group of SVF cell induced to differentiation into white adipocytes on day 8, including the lipogenic transcription factor *C/EBPα*, the lipogenic gene *ACACA*, and the rate-limiting enzymes for fat synthesis, *DGAT1* and *DGAT2*. Lipid content and the gene expression level are represented as mean, with error bars indicating the standard error of the means (mean ± SEM); *p* > 0.05, * *p* < 0.05, ** *p* < 0.01, and *** *p* < 0.001.

Six SCFAs were identified as being significantly and positively correlated with porcine adiposity. It was hypothesized that the lower adiposity of KI pigs may be caused by a reduction in the content of SCFAs in their intestines. However, the effect of SCFAs on porcine fat deposition remains unknown. It was therefore decided that caproic acid, which is the most different in the feces of WT and KI pigs (except for isovaleric acid), should be the subject of further investigation with a view to establishing its role in regulating lipid deposition in porcine SVF cells. SVF cells were isolated from the subcutaneous adipose tissue of Bama pigs and added to a medium containing varying concentrations of sodium caproate salt. This induced the differentiation of SVF cells into mature adipocytes, allowing for an assessment of the lipogenic efficiency of this differentiation process on the formation of white adipocytes (Figure 4A). Following an eight-day period of induced differentiation, the SVF was observed to successfully differentiate into mature adipocytes. The treatment of sodium caproate at a concentration of 0.1 mM was found to significantly enhance the efficiency of SVF differentiation into mature adipocytes (Figure 4B,C, *p* < 0.01). Conversely, sodium caproate at a concentration of 3 mM was observed to significantly reduce the efficiency of SVF differentiation into white adipocytes (Figure 4B,C, *p* < 0.01) [21]. Therefore, the alterations in the expression of the *FFAR2* and *FFAR4* genes were identified following the administration of varying concentrations of sodium caproate. The findings demonstrated that sodium caproate treatment could stimulate the expression of *FFAR4* (Figure 4D). The aforementioned results indicated that sodium caproate could promote the differentiation of SVF cells to mature adipocytes by activating the expression of the *FFAR4* gene, with the 0.1 mM concentration demonstrating the most pronounced effect.

## 4. Discussion

Leanness is an important indicator of pig growth and farrowing time. Consequently, breeding low-fat-rate pig breeds is now one of the goals of pig breeding. This is achieved by identifying candidate genes that regulate lipid metabolism in pigs. Furthermore, the excavation and identification of the intestinal probiotics and prebiotics associated with pig fat deposition represents another means of regulating pig growth from the perspective of energy intake [22].

In a previous study, we employed CRISPR/Cas9 technology to generate genetically modified pigs with the adipose tissue-specific expression of the *UCP1* gene. The KI pigs displayed a reduced fat percentage and an increased lean mass compared to the WT pigs [1]. The genetic background of the KI pigs is consistent with that of the WT pigs, with the exception of differences in adipose tissue *UCP1* gene expression. This makes them an appropriate model for screening probiotics and prebiotics. Short-chain fatty acids (SCFAs) constitute a group of saturated fatty acids with a chain length of one to six carbon atoms. They are primarily produced by gut microorganisms that ferment unconsumed fibre in the diet, providing energy to intestinal epithelial cells and supplying approximately 10% of the host’s energy each day [23]. The proportion of acetate, butyrate, and propionate in the SCFAs produced in the host intestine was high, accounting for approximately 90% of the total SCFAs [24]. The principal components of the seven SCFAs identified were acetic acid, butyric acid, and propionic acid, collectively accounting for approximately 80% of the total SCFAs. The production of SCFAs by the gut can regulate the host’s energy homeostasis and contribute to the development of the host’s obese phenotype [25]. A number of studies have indicated that individuals with a higher body mass index (BMI) have a greater concentration of SCFAs in their feces than those with a lower BMI [23]. Notably, this difference is not attributed to variations in dietary intake or the absorption of SCFAs [26]. The findings of our study align with the observation that the fat percentage was markedly lower in KI pigs compared to WT pigs. Additionally, the total amount of SCFAs and the concentration of seven SCFAs in the feces of KI pigs were found to be significantly reduced in comparison to WT pigs (Figure 1). SCFAs are produced by gut microbes that ferment dietary fibres that are not easily digested by the host. The fecal microbial composition of the KI pigs in this study differed significantly from that of the WT pigs, which may be the primary factor contributing to the observed differences in SCFAs. It is commonly reported in the literature that butyric acid production is typically attributed to *Firmicutes* [27]. The butyric acid content of the feces of the KI pigs was found to be significantly lower than that of the wild-type pigs, which is consistent with the hypothesis that the relative abundance of the thick-walled phylum is lower in the former than in the latter at six months of age. The results of the correlation analysis indicated that the abundance of *Streptococcus* spp. was significantly and positively correlated with the content of differential SCFAs and also positively correlated with adiposity. This suggests that *Streptococcus* spp. may be involved in the production of SCFAs in the intestinal tracts of pigs in the KI, which is consistent with the production of acetic acid SCFAs by *Streptococcus* spp. as reported in the literature [28]. Nevertheless, the identity of the predominant species of *Streptococcus* awaits confirmation. It has been documented in the scientific literature that SCFAs not only provide energy to the host organism but are also pivotal signal molecules that function by binding to the G protein-coupled receptors free fatty acid receptor 2 (*FFAR2*) and free fatty acid receptor 4 (*FFAR4*). These receptors are expressed in the intestinal mucosa, immune cells, liver, and adipose tissue [29]. It has been demonstrated that the expression of *FFAR4* in adipose tissue is markedly elevated in individuals with obesity in comparison to those with a normal BMI. Furthermore, a detrimental variant of *FFAR4* (p.R270H) has been linked to elevated fasting glucose levels and an increased risk of obesity. This variant has also been observed to play a role in the development of obesity in both human and murine models [30,31]. Concurrently, some studies have indicated that SCFAs can stimulate *FFAR2*, thereby enhancing energy uptake in white adipose tissue [32]. Nevertheless, it has been documented in the scientific literature that acetate exerts anti-lipolytic effects through the inhibition of HSL phosphorylation in human pluripotent adipose tissue-derived stem cells, which is mediated by FFARs [33]. The present study revealed that the concentration of SCFAs in the feces of KI pigs was markedly lower than that of WT pigs. Furthermore, a significant positive correlation was observed between the SCFA content and porcine adiposity. However, the impact of SCFAs on porcine fat deposition remains unclear. Accordingly, we examined the impact of caproic acid, which exhibited the most pronounced variation in KI and WT pigs, on the in vitro lipogenesis of SVF cells. The findings indicated the administration of sodium caproate at a concentration of 0.1 mM. The differentiation efficiency of SVF to mature adipocytes was significantly increased by 0.1 mM sodium caproate; the expression of genes related to lipogenesis was increased as well. However, the treatment of sodium caproate at a concentration of 3 mM was ineffective, which may be due to the fact that the sodium caproate at a concentration of 0.1 mM promotes SVF proliferation and lipid synthesis, but the 3 mM concentration of sodium caproate had a certain toxic effect on cells and promoted apoptosis. Nevertheless, the treatment of SVF cells with either 0.1 mM or 3 mM concentrations of caproic acid sodium salt was observed to significantly upregulate *FFAR4* gene expression, while having no effect on FFAR2. This suggests that caproic acid sodium salt may regulate the differentiation of SVF cells to white adipocytes and lipogenesis through *FFAR4* activation but not FFAR2. This is consistent with the findings that high concentrations of caproic acid are associated with type 2 diabetes and obesity [34]. Furthermore, this is consistent with the observation of low concentrations of SCFAs and low adiposity in the feces of KI pigs. Nevertheless, further validation and mechanistic elucidation at the individual level are required to confirm the mechanisms by which the high expression of the UCP1 gene in adipose tissue in KI pigs regulates the reduction in *Streptococcus* spp. abundance and produces SCFAs. Additionally, the role of SCFAs in the regulation of adipose tissue lipogenesis remains to be elucidated. The present study sought to elucidate the compositional differences in SCFAs and intestinal flora between KI and WT pigs. It revealed a notable decline in the concentration of SCFAs in the feces of KI pigs, which may be linked to a reduction in the prevalence of *Streptococcus* spp.

Furthermore, the study demonstrated the efficacy of SCFAs in promoting the differentiation of SVF cells into mature adipocytes through the activation of *FFAR4* gene expression. This study not only adds to our further knowledge of the molecular mechanisms underlying the low-fat phenotype of KI pigs but also provides a new perspective and scientific basis for the development of probiotics and prebiotics that modulate fat deposition in pigs.

## 5. Conclusions

The concentration of SCFAs in feces was found to be significantly lower in KI pigs compared to WT pigs, which may be associated with a reduction in the abundance of *Streptococcus* spp. The differentiation of porcine SVF cells to mature adipocytes was significantly enhanced by 0.1 mM caproate, which was observed to activate the *FFAR4* gene.

## Figures and Tables

**Table 1:** The content of fecal SCFAs of WT and KI pigs.

Species	WT (μg/mL)	KI (μg/mL)	*p*-Value	Fold Change
Acetic acid	61.61 ± 0.65	51.91 ± 1.43	4.37 × 10^−4^	1.19
Methylacetic acid	48.37 ± 1.37	37.45 ± 1.03	3.89 × 10^−4^	1.29
Isobutyric acid	14.41 ± 0.57	8.34 ± 0.40	1.12 × 10^−4^	1.73
Butyrate acid	27.41 ± 0.99	25.02 ± 0.98	4.09 × 10^−2^	1.10
Isovaleric acid	16.12 ± 0.66	7.95 ± 0.35	4.53 × 10^−5^	2.03
Valeric acid	19.44 ± 0.76	11.22 ± 0.47	9.09 × 10^−5^	1.73
Caproic acid	0.40 ± 0.02	0.22 ± 0.01	4.09 × 10^−5^	1.84

*p* < 0.05 indicates a significant difference, while *p* > 0.05 indicates no significant difference.

## Data Availability

The data that support the findings of this study are available from the corresponding author upon reasonable request.

## Supplementary Materials

The following supporting information can be downloaded at: https://www.mdpi.com/article/10.3390/vetsci12020102/s1, Table S1: QPCR Primer sequences.
