# Supplementary material for: Differential Analysis of Fecal SCFAs and Their Contribution to Adipogenesis in UCP1 Knock-In Pigs
Source: Vet Sci. 2025 Feb 1;12(2):102. doi: 10.3390/vetsci12020102 (PMC11860427; doi:10.3390/vetsci12020102)
Supplement: Supplementary file 1 [file vetsci-12-00102-s001.zip › vetsci-3330044-supplementary.pdf]

Table S1. Primer sequences

Genes	Primer sequences（5′-3′）
18s-F	GTAACCCGTTGAACCCCATT
18s-R	CCATCCAATCGGTAGTAGCG
C/EBPα-F	GGCCAGCACACACACATTAGA
C/EBPα-R	CCCCCAAAGAAGAGAACCAAG
ACACA-F	CGTGCAATCCGGTTTGTTGT
ACACA-R	TGTTGTTGTTTGGGCCTCCT
DGAT1-F	CCCACCATCCAGAACTCCAT
DGAT1-R	CGGTCTCCAAACTGCATGAG
DGAT2-F	CCCTCATAGCTGCCTACTCC
DGAT2-R	GAGGAAAGACAGGACCCACT
FFAR4-F	TCGCTCTCATCTGGGGCTAT
FFAR4-R	TGATCGGGCTCCACATGATG

* Primer sequences for pigs.
